# Clearance of multiple antibiotic-resistant coagulase-negative staphylococci is selectively associated with higher circulating α-melanocyte stimulating hormone in patients evaluated for chronic inflammatory response syndrome

**DOI:** 10.3389/fendo.2026.1728408

**Published:** 2026-04-17

**Authors:** Margaret DiTulio, Christian A. Navarro-Torres

**Affiliations:** 1 Regenix Healing LLC - Atkinson, New Hampshire, NH, United States; 2 Chronic Inflammatory Response Syndrome (CIRS) Research Foundation - Atkinson, New Hampshire, NH, United States

**Keywords:** alpha-melanocyte stimulating hormone, coagulase-negative staphylococci, nasal microbiome, sinonasal colonization, chronic inflammation, Chronic Inflammatory Response Syndrome, neuroendocrine signaling

## Abstract

**Introduction:**

Neuroimmune regulatory peptides play central roles in coordinating inflammatory, metabolic, and mucosal immune processes in humans. Among these, α-melanocyte stimulating hormone (α-MSH), a proopiomelanocortin-derived peptide, has been implicated in modulation of cytokine signaling, epithelial barrier function, and pain processing. However, determinants of circulating α-MSH levels in chronic inflammatory states remain incompletely characterized in human clinical populations. Persistent sinonasal colonization with multiple antibiotic-resistant coagulase-negative staphylococci (MARCoNS) has been reported in some cohorts presenting with environmentally associated multisystem illness, described in some clinical settings as Chronic Inflammatory Response Syndrome (CIRS). Yet its relationship to systemic neuroendocrine biomarkers has not been quantitatively examined.

**Methods:**

This retrospective observational cohort study examined whether follow-up MARCoNS culture status was associated with longitudinal trajectories of α-MSH, compared with two additional biomarkers commonly assessed in this population: matrix metallopeptidase-9 (MMP-9) and vasoactive intestinal polypeptide (VIP). A total of 188 adult patients evaluated within a CIRS-informed clinical framework at a single clinical site were included. Each participant contributed two timepoints of laboratory data for α-MSH, MMP-9, and VIP, along with MARCoNS culture results.

**Results:**

Across the full cohort, α-MSH increased by an estimated 10 pg/mL, MMP-9 decreased by 398 ng/mL, and VIP increased by 20 pg/mL between baseline and follow-up. Mixed-effects modeling revealed a significant timepoint-by-MARCoNS interaction for α-MSH, such that patients who were MARCoNS-negative at follow-up exhibited higher circulating α-MSH levels compared with those who remained positive. In contrast, no corresponding MARCoNS interaction was observed for VIP or MMP-9.

**Discussion:**

These findings provide quantitative evidence that follow-up MARCoNS culture status is selectively associated with α-MSH trajectories in this retrospective cohort, supporting further prospective investigation into potential links between persistent bacterial nasal colonization and neuroendocrine-immune biomarkers in multisystem chronic illness.

## Introduction

Neuroimmune regulatory peptides play a central role in maintaining homeostasis at the interface of inflammation, metabolism, and mucosal immunity. Among these, α-Melanocyte Stimulating Hormone (α-MSH), a proopiomelanocortin (POMC)-derived neuropeptide, has been implicated in modulation of inflammatory cytokine signaling, epithelial barrier function, antimicrobial activity, metabolic regulation, and nociceptive processing across preclinical studies (1–14). Despite extensive mechanistic characterization, the determinants of circulating α-MSH levels in human chronic inflammatory states remain incompletely defined, with limited human biomarker data available to date (15). The present study examines whether persistent sinonasal bacterial colonization is selectively associated with longitudinal shifts in circulating neuroendocrine biomarkers, with particular focus on α-MSH, within a clinically characterized cohort of patients presenting with environmentally associated multisystem illness.

Chronic multisystem symptom presentations following environmental or microbial exposures have been described across multiple clinical and public health constructs, including Sick Building Syndrome (SBS), myalgic encephalomyelitis/chronic fatigue syndrome (ME/CFS), and related exposure-associated illness profiles (16–24). These syndromes frequently include fatigue, cognitive symptoms, pain, and inflammatory biomarkers, although their mechanistic underpinnings remain under active investigation. Within some clinical settings, Chronic Inflammatory Response Syndrome (CIRS) has been proposed as an operational framework for characterizing a subset of patients with persistent multisystem symptoms following environmental or microbial exposures, particularly in the context of water-damaged buildings (25–27). Reports within this literature describe impaired visual contrast sensitivity, innate immune overactivation, and neuroendocrine abnormalities, including reduced circulating α-MSH (28–31). While the nosological boundaries of such constructs remain empirically under investigation, they provide clinically characterized cohorts in which mechanistic biomarker relationships can be systematically examined.

One microbial factor frequently evaluated within cohorts evaluated for CIRS is persistent sinonasal colonization with multiple antibiotic-resistant coagulase-negative staphylococci (MARCoNS). CoNS are a diverse group of Gram-positive, facultative anaerobic cocci that constitute a major component of the normal human skin. Historically regarded as benign commensals in immunocompetent hosts, CoNS have increasingly drawn attention due to their capacity to adopt opportunistic behaviors under conditions of host vulnerability or microbial imbalance (32). Unlike *S. aureus*, CoNS lack the enzyme coagulase, which contributes to their traditionally lower virulence. However, they may exhibit opportunistic virulence traits through sophisticated immune evasion strategies, including biofilm formation, antibiotic resistance, and the release of putative membrane-damaging toxins (33–35). Further, CoNS can employ passive defense mechanisms that can inactivate antimicrobial peptides (AMPs), which may be critical for maintaining a healthy epithelial microflora (36).

Preliminary clinical observations with patients evaluated for environmentally related multisystem illness have documented an association between MARCoNS sinonasal colonization and reduced circulating α-MSH levels (37, 38). Other observational work has further reported associations between CoNS colonization and chronic orofacial pain in patients with chronic pain and chronic fatigue syndromes (39, 40, 46). Given the documented pain-modulating properties of exogenous α-MSH in preclinical inflammatory models (8), these clinical associations raise the possibility that persistent mucosal CoNS colonization may in fact intersect with melanocortin-regulated neuroimmune pathways.

The present retrospective observational cohort study (N = 188) examines whether follow-up MARCoNS culture status (positive vs negative) is associated with longitudinal trajectories of circulating α-MSH, in comparison with vasoactive intestinal polypeptide (VIP) and Matrix metalloproteinase-9 (MMP-9), two biomarkers commonly reported as abnormal in this clinical population. Timepoints were defined chronologically as pre-treatment baseline (T1) and post-treatment follow-up (T2). We hypothesized that MARCoNS-negative status at follow-up would be selectively associated with higher α-MSH levels, but not MMP-9 or VIP.

## Materials and methods

### Patients

De-identified retrospective clinical data from 188 adult patients evaluated and treated for CIRS were collected at a single outpatient site (Regenix Healing, LLC). Patient characteristics are summarized in **Table 1**. Diagnosis was determined clinically using published criteria and a standard differential evaluation process (15), with consideration of alternative explanations including autoimmune disease, atypical infections, neurodegenerative disorders, and neuropsychiatric conditions.

**Table 1:** Patient characteristics.

Characteristic	Mean/percentage (range/count)	N
Age (years)	51 (18-80)	188
Sex Male Female	22% (41) 78% (147)	188
CIRS Treatment Group Group 1 Group 2	54% (102) 46% (86)	188
MARCoNS+	50% (94)	188

**Figure 1:**
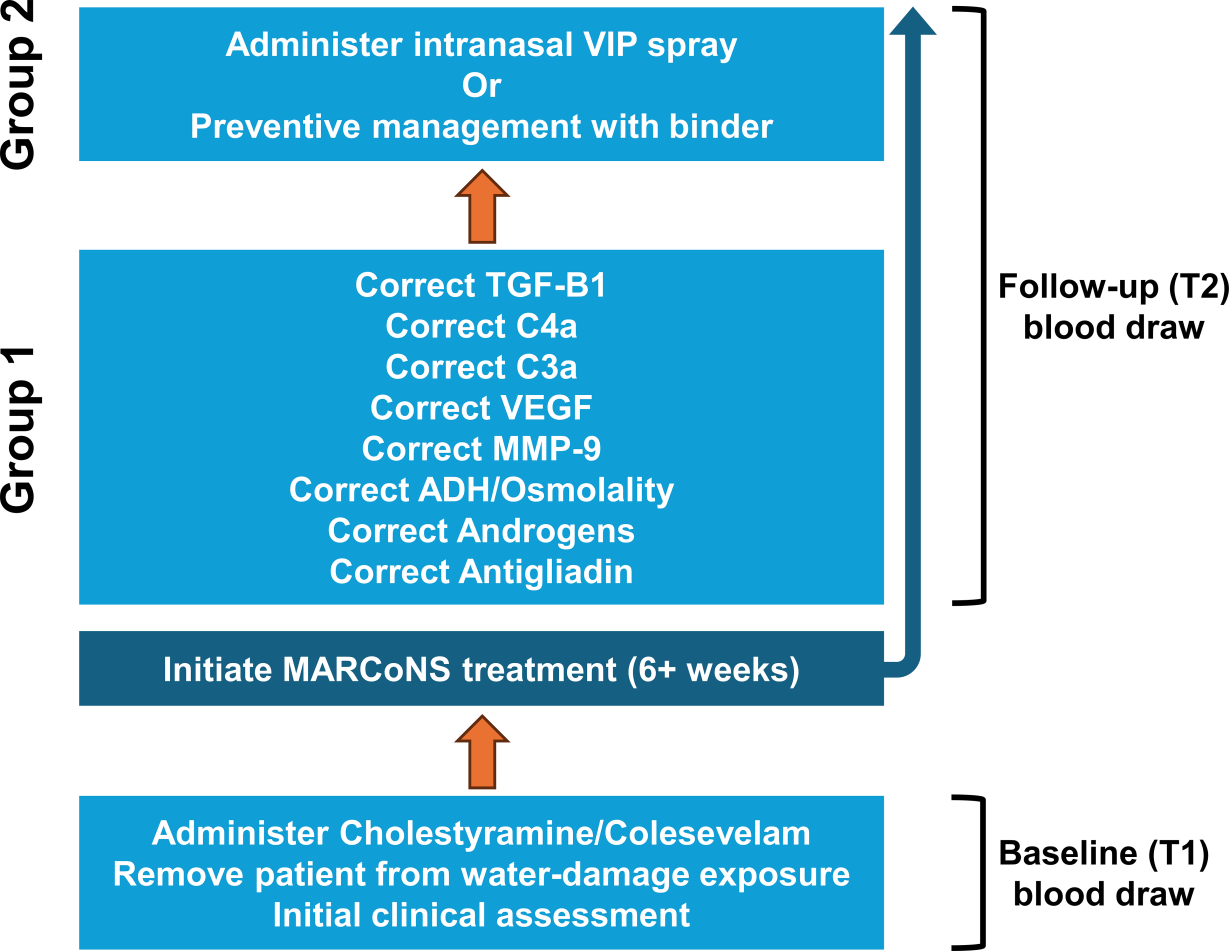
Schematic overview of the clinical treatment sequence used in the retrospective cohort from which study data were derived. All patients underwent baseline evaluation and initial biomarker assessment at Timepoint 1 (T1), followed by environmental avoidance and binder therapy (e.g., cholestyramine/colesevelam) and initiation of MARCoNS-directed treatment (≥6 weeks). Patients were subsequently managed in one of two clinical stages at follow-up (Timepoint 2; T2): Group 1, representing individuals still undergoing correction of inflammatory and neuroendocrine abnormalities, and Group 2, representing individuals who had progressed to intranasal vasoactive intestinal polypeptide (VIP) therapy or preventive maintenance. MARCoNS culture status at T2 (positive vs. negative) served as the primary grouping variable in longitudinal analyses examining trajectories of α-MSH, VIP, and MMP-9 across T1 and T2. This schematic is provided for clinical context within a retrospective observational design.

Group 1, patients still undergoing initial CIRS episode at follow-up (T2); Group 2, patients who completed initial treatment or initiated exogenous VIP therapy at follow-up (T2; see **Figure 1** for an illustration); MARCoNS+, patients with a positive nasal culture for MARCoNS at follow-up (T2).

All patients designated with CIRS met the clinic’s standard diagnostic criteria based on published framework components (5, 41). This included: 1) positive for ≥8 of 13 symptom clusters (see **Supplementary Table 1** in **Supplementary Materials**); 2) documented exposure history consistent with biotoxin-associated environments (most commonly water-damaged buildings with suspected fungal and/or bacterial contamination); and 3) laboratory abnormalities reflecting innate immune activation and neuroendocrine dysregulation. Biomarker domains evaluated clinically included complement activation (C3a, C4a), inflammatory and matrix remodeling innate immune markers (MMP-9, TGF-β1), vascular regulation (VEGF), and regulatory hormone abnormalities (α-MSH, ACTH, VIP, ADH/osmolality). Diagnosis was determined following clinical exclusion of alternative etiologies through a differential process that commonly included autoimmune disease, atypical infections (e.g., Lyme disease), neurodegenerative conditions, and primary neuropsychiatric disorders.

Inclusion criteria for the present study were: age ≥18 years, clinical evaluation for CIRS by the clinic director, availability of two MARCoNS nasal culture results obtained at least six weeks apart, two corresponding plasma α-MSH measurements, and an initial positive MARCoNS culture at baseline (T1). Patients were excluded if they were under 18 years of age or lacked the required paired culture and biomarker measurements.

As shown in **Table 1**, the cohort was predominantly composed of middle-aged females. At the time of assessment, patients had undergone environmental avoidance or remediation efforts and were actively engaged in treatment (**Figure 1**). Approximately half of participants were classified as Group 1, representing individuals undergoing treatment for an initial CIRS episode. Group 2 included patients who had completed prior CIRS treatment and were either receiving intranasal VIP for residual clinical indications (29) or engaged in preventive management strategies, including intermittent use of binding agents during periods of increased re-exposure risk.

### Blood and nasal culture collection

Blood samples for circulating α-MSH, MMP-9, and VIP were obtained under non-fasting conditions and processed through LabCorp using standardized clinical laboratory procedures. According to the laboratory assay specifications, α-MSH and VIP were measured in EDTA plasma using radioimmunoassay methodology, and MMP-9 was measured using a validated immunoassay platform as described by the reference laboratory (LabCorp test codes: α-MSH [010421], VIP [010397], MMP-9 [500124]). Per assay specifications, specimens were collected and handled in accordance with LabCorp pre-analytical requirements, including immediate processing and frozen shipment where applicable. The lower reportable limit for the α-MSH assay was 8 pg/mL, which was treated as the assay detection floor in interpretation. Values reported as “<8 pg/mL” were treated as left-censored and were recorded as 7 pg/mL for analysis. We report the proportions of values below and at the lower reportable limit at each timepoint in the Results.

Nasal culture specimens were obtained and submitted to Microbiology DX, a CLIA-certified microbiology laboratory specializing in nares bacterial culture testing. Microbiology DX performed aerobic culture of nasal swabs using standard microbiological techniques to isolate and identify bacterial species, including coagulase-negative *Staphylococcus* (CoNS). When bacterial isolates were obtained, antibiotic susceptibility testing was performed against a panel that included quinolones, erythromycin, clindamycin, rifampicin, gentamicin, penicillin-G, oxacillin/methicillin, synercid, trimethoprim-sulfamethoxazole, tigecycline, vancomycin, and tetracycline.

In clinical diagnostic laboratories, susceptibility testing is typically performed using standardized phenotypic methods that assess bacterial growth inhibition in the presence of antimicrobial agents (e.g., disk diffusion or gradient diffusion assays), yielding categorical interpretations (susceptible, intermediate, or resistant) for each antibiotic tested. A positive MARCoNS designation in this study was defined as the presence of a CoNS isolate exhibiting resistance to two or more antimicrobial agents on the susceptibility profile. A negative MARCoNS designation was defined as either: (a) absence of CoNS growth on culture, or (b) isolation of CoNS with resistance to no more than one antimicrobial agent. These operational definitions align with the reporting framework provided by the reference laboratory.

According to the Microbiology DX’s standard operating procedure, nasal swabs were cultured aerobically on blood agar (BAP), colistin-nalidixic acid (CNA) blood agar, and MacConkey agar to support recovery of both gram-positive and gram-negative organisms. Colonies consistent with CoNS were identified based on morphology (white, moist colonies), Gram stain demonstrating gram-positive cocci, and phenotypic testing including catalase positivity and absence of coagulase activity using a rapid latex agglutination assay. Antibiotic susceptibility testing was subsequently performed using a semi-automated phenotypic analyzer (Vitek 2 platform) against a multi-drug panel. In accordance with the laboratory’s reporting framework, a MARCoNS designation was assigned when a CoNS isolate demonstrated resistance to at least two antimicrobial agents, or one resistant and one intermediate result, on the susceptibility profile. Quality control procedures, including use of positive and negative controls and daily documentation, were performed in accordance with CLIA laboratory standards.

### Procedure

**Figure 1** summarizes the clinical management protocol applied within this practice for patients evaluated under a Chronic Inflammatory Response Syndrome (CIRS) framework. In this cohort, patients presented with chronic multisystem symptom profiles in the context of suspected exposure to water-damaged indoor environments with potential microbial burden. Accordingly, an initial management step involved avoidance or remediation of the suspected exposure environment when feasible.

As part of the broader clinical protocol, patients commonly received bile acid sequestrants (colesevelam or cholestyramine), which have been evaluated in prior clinical studies of symptom-based illness associated with water-damaged building exposure, including treatment and re-exposure designs (31). These interventions were administered as part of routine care within this retrospective observational cohort.

Following this step, all patients included in the present cohort had positive baseline MARCoNS nasal cultures and were therefore treated with a topical intranasal regimen for a minimum of six weeks. Some patients continued MARCoNS-directed therapy beyond this initial interval depending on clinical response, organism burden, and antibiotic resistance patterns. However, the exact elapsed time between laboratory collections varied in routine clinical care and could not be quantified from the available extract. Treatment approaches varied and included compounded nasal formulations containing agents such as xylitol, EDTA, colloidal silver, and grapefruit seed extract, administered individually or in combination.

Baseline (T1) blood draws were obtained prior to initiation of MARCoNS-directed therapy, whereas follow-up (T2) blood draws were obtained during later stages of the clinical protocol, after completion of at least the initial six-week intranasal treatment interval. Because care was delivered as part of a multi-component observational treatment program, the present analyses focus on associations with follow-up MARCoNS culture status rather than causal attribution to any single intervention component.

### Statistical analysis

To evaluate changes in biomarker levels and their associations with bacterial colonization, we conducted three separate linear mixed-effects models (LMMs) using the lme4 software package, version 1.1-35.1 (42), in the R programming environment (43). The core models included one of the following blood biomarkers as the dependent variable: α-MSH, MMP-9, or VIP.

Each of these primary models included the following fixed effects: 1) Timepoint, a within-subjects categorical factor indicating whether the biomarker measurement was taken at baseline (T1) or at follow up (T2); 2) MARCoNS Status, a between-subjects categorical factor indicating the presence or absence of MARCoNS in the nasal passages at T2; and 3) two covariates, age and biological sex. Both Timepoint and MARCoNS Status were allowed to interact with one another. A random intercept for each patient was included to account for repeated measures and inter-individual variability. No random slopes were specified due to model conversion warnings. This model setup resulted in the following R codes:

α-MSH ~ Age + Sex + MARCoNS Status*Timepoint + (1|Patient).

MMP-9 ~ Age + Sex + MARCoNS Status*Timepoint + (1|Patient).

VIP ~ Age + Sex + MARCoNS Status*Timepoint + (1|Patient).

In addition to the primary mixed-effects models, baseline comparability between patients who were MARCoNS-positive versus MARCoNS-negative at follow-up was evaluated using Welch’s independent-samples t-tests for continuous demographic and biomarker variables and chi-square tests for categorical variables (sex). These analyses were included to assess potential baseline imbalances prior to longitudinal modeling.

We also conducted sensitivity analyses to evaluate robustness to baseline biomarker differences and potential regression-to-the-mean effects using baseline-adjusted ANCOVA-style regression modelling. We included follow-up α-MSH (T2) as the dependent variable while adjusting for baseline α-MSH (T1). This model additionally included age, sex, and baseline (T1) levels of VIP and MMP-9 as covariates. Finally, we performed sensitivity analyses excluding patients who initiated exogenous VIP therapy to assess potential treatment-related confounding. These secondary analyses were performed to strengthen inference given the retrospective observational design.

For mixed-effects and ANCOVA-style regression models, categorical predictors were centered using sum-to-zero contrast coding (± 0.5) and continuous covariates were z-standardized to aid interpretability of model coefficients and intercepts. Denominator degrees of freedom were estimated using the Satterthwaite approximation as implemented in the lmerTest package (version 3.1-3) (44). When significant interactions were observed, follow-up comparisons (simple effects) were obtained by reparameterizing the model with alternative reference coding to estimate conditional effects at each level of the interacting factor. These follow-up contrasts are reported in the text for interpretive clarity. Importantly, such reparameterization does not change the underlying model fit or variance structure, but provides equivalent estimates expressed relative to different reference points (45).

## Results

Baseline demographic and biomarker characteristics at T1 were comparable between patients who were MARCoNS-positive versus MARCoNS-negative at T2 (**Table 2**). No significant baseline differences were observed between groups in circulating α-MSH, VIP, or MMP-9, and mean age was also similar across MARCoNS status groups. Sex distribution likewise did not differ significantly by follow-up MARCoNS status (**Table 3**), with no evidence of association in a chi-square test. Collectively, these findings support baseline comparability between groups and reduce concern that subsequent longitudinal differences reflect pre-existing demographic or biomarker imbalances rather than changes associated with follow-up colonization status.

**Table 2:** Baseline (T1) comparisons by MARCoNS Status at follow-up (T2).

	MARCoNS+	MARCoNS−	*t*	*p*
	Mean (SD) N			
α-MSH (pg/mL) at T1	8.44 (2.56) N=94	8.62 (4.34) N=94	-0.35	.726
MMP-9 (mg/mL) at T1	736.92 (325.62) N=93	759.48 (344.59) N=91	-0.46	.648
VIP (pg/mL) at T1	20.20 (8.58) N=77	20.37 (8.34) N=72	-0.12	.905
Age (years)	44.30 (10.92) N=94	44.70 (11.03) N=94	-0.25	.801

Welch two-sample t-tests were used; MARCoNS+- = patients with a positive or negative nasal culture for MARCoNS at T2.

**Table 3:** Sex distribution by MARCoNS Status at follow-up (T2).

	N	Females (n)	Female %	χ²	df	*p*
MARCoNS+	94	76	80.9%	0.78	1	0.380
MARCoNS−	94	71	75.5%			

MARCoNS±, patients with a positive or negative nasal culture for MARCoNS at T2.

At T1, 144 of 188 participants (76.6%) had α-MSH values at or below the assay detection boundary (<8 pg/mL coded as 7 pg/mL; n=143; 8 pg/mL; n=1). At T2, 24 participants (12.8%) remained at or below the detection boundary (20 coded as 7; 4 coded as 8). The proportion at the detection limit at T2 did not differ between MARCoNS-negative and MARCoNS-positive groups (12/94 in each group).

**Table 4** presents the raw mean circulating levels of α-MSH, MMP-9, and VIP at baseline (T1) and follow-up (T2), stratified by follow-up MARCoNS culture status (MARCoNS-positive vs. MARCoNS-negative). Consistent with these descriptive trends, mixed-effects models adjusting for age and sex revealed significant main effects of Timepoint across all three biomarkers (**Tables 5**–**7**). Specifically, α-MSH increased by an estimated 10 pg/mL from T1 to T2, VIP increased by approximately 20 pg/mL, and MMP-9 decreased by approximately 398 ng/mL over the same interval. Together, these findings indicate broad longitudinal shifts in neuroendocrine and inflammatory biomarkers in this clinical cohort.

**Table 4:** Changes in mean plasma biomarker levels stratified by MARCoNS culture status at follow-up.

	MARCoNS+ T1	MARCoNS+ T2	MARCoNS− T1	MARCoNS− T2
	Mean (SD) N			
α-MSH (pg/mL)	8.44 (2.56) N=94	16.63 (5.87) N=94	8.62 (4.34) N=94	20.10 (6.79) N=94
MMP-9 (ng/mL)	736.92 (325.62) N=93	339.43 (157.36) N=93	759.48 (344.59) N=91	365.36 (185.61), N=91
VIP (pg/mL)	20.20 (8.58) N=77	39.40 (17.53) N=77	20.37 (8.34) N=72	41.22 (18.62), N=72

T1, Timepoint 1; T2, Timepoint 2.

**Table 5:** Model estimates for α-MSH.

Fixed Effects	Estimate	*SE*	df	*t*	*p*	95% CI
Intercept	13.69	0.48	184	28.53	<.001	[12.75, 14.63]
Age	-0.87	0.40	184	-2.18	.031	[-1.65, -0.09]
Sex	-0.81	0.96	184	-0.84	.403	[-2.69, 1.08]
MARCoNS Status	1.90	0.79	184	2.39	.018	[0.34, 3.45]
Timepoint	10.14	0.60	186	16.77	<.001	[8.96, 11.33]
MARCoNS × Timepoint	4.06	1.21	186	3.35	<.001	[1.69, 6.43]
Random Effects	Variance	*SD*				
Intercept Patient	12.27	3.50				
Residual	34.39	5.86				

df computed using Satterthwaite approximation (lmerTest). Confidence intervals are Wald 95% intervals from confint(). Random effects are reported as variance components for the patient-level random intercept and residual error. Age was z-standardized and categorical predictors were sum-coded (± 0.5), such that the intercept represents the grand mean biomarker level across groups and timepoints.

**Table 6:** Model estimates for MMP-9.

Fixed effects	Estimate	*SE*	df	*t*	*p*	95% CI
Intercept	557.87	19.82	179	28.15	<.001	[519.03, 596.70]
Age	-29.69	16.58	179	-1.79	.075	[-62.19, 2.80]
Sex	-19.51	39.73	179	-0.49	.624	[-97.37, 58.36]
MARCoNS Status	-40.30	32.82	179	-1.23	.221	[-104.62, 24.02]
Timepoint	-397.35	22.14	181	-17.94	<.001	[-440.75, -353.95]
MARCoNS × Timepoint	24.41	44.29	181	0.55	.582	[-62.39, 111.22]
Random Effects	Variance	*SD*				
Intercept Patient	26622	163.2				
Residual	44856	211.8				

df computed using Satterthwaite approximation (lmerTest). Confidence intervals are Wald 95% intervals from confint(). Random effects are reported as variance components for the patient-level random intercept and residual error. Age was z-standardized and categorical predictors were sum-coded (± 0.5), such that the intercept represents the grand mean biomarker level across groups and timepoints.

**Table 7:** Model estimates for VIP.

Fixed effects	Estimate	*SE*	df	*t*	*p*	95% CI
Intercept	30.20	1.15	145	26.20	<.001	[27.94, 32.46]
Age	2.62	0.97	145	2.70	.0078	[0.72, 4.52]
Sex	-0.08	2.31	145	-0.03	.974	[-4.60, 4.44]
MARCoNS Status	1.24	1.92	145	0.65	.519	[-2.52, 4.99]
Timepoint	19.66	1.26	147	15.65	<.001	[17.20, 22.12]
MARCoNS × Timepoint	1.37	2.51	147	0.55	.586	[-3.55, 6.29]
Random Effects	Variance	*SD*				
Intercept Patient	77.77	8.82				
Residual	117.46	10.64				

df computed using Satterthwaite approximation (lmerTest). Confidence intervals are Wald 95% intervals from confint(). Random effects are reported as variance components for the patient-level random intercept and residual error. Age was z-standardized and categorical predictors were sum-coded (± 0.5), such that the intercept represents the grand mean biomarker level across groups and timepoints.

**Figure 2:**
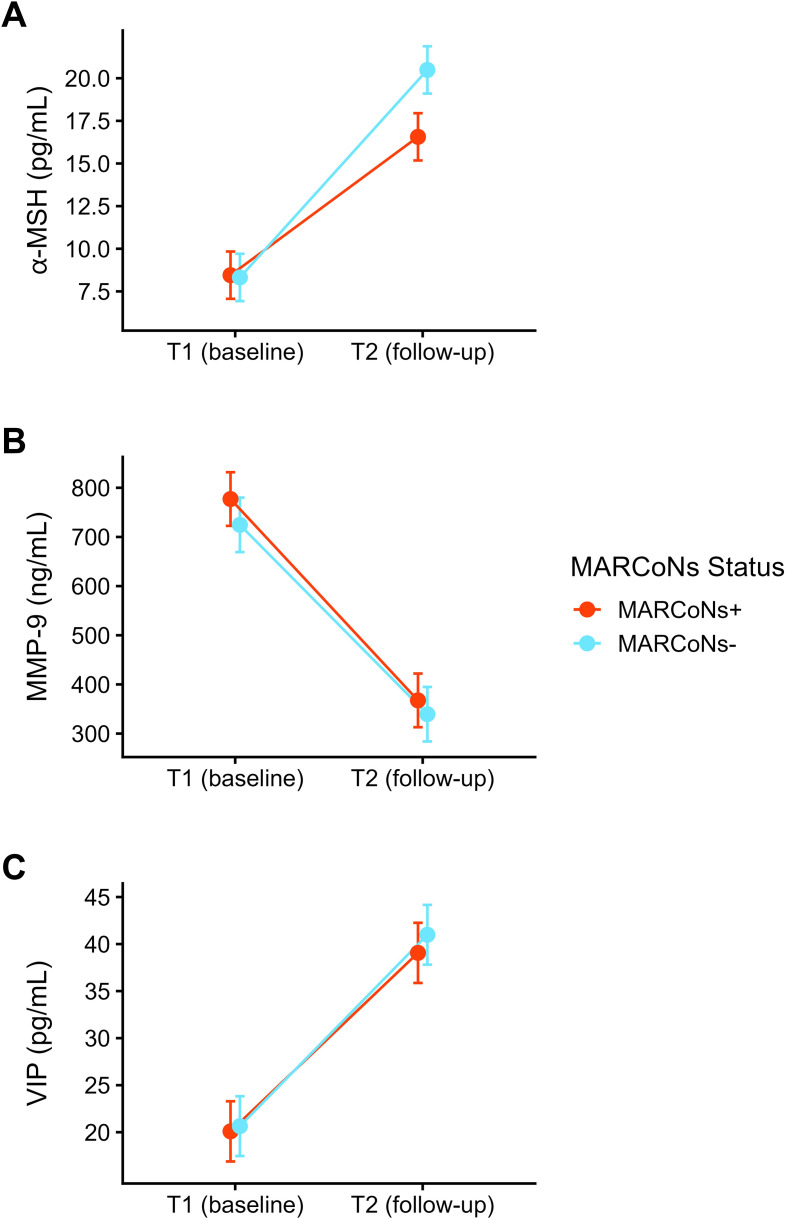
Model-estimated plasma levels of three biomarkers, α-MSH **(A)**, MMP-9 **(B)**, and VIP **(C)**, measured at two clinical timepoints. Timepoint 1 (T1) represents a baseline measurement before beginning MARCoNS treatment, and Timepoint 2 (T2) represents a follow-up measurement obtained at least 6 weeks after starting MARCoNS treatment. Biomarker trajectories are shown separately for patients who tested MARCoNS-positive versus MARCoNS-negative at T2. Values represent estimated marginal means from linear mixed-effects models adjusting for age and sex, with error bars indicating 95% confidence intervals. A significant MARCoNS Status × Timepoint interaction was observed for α-MSH **(A)**, indicating greater longitudinal increases among MARCoNS-negative patients, whereas no such interaction was observed for MMP-9 **(B)** or VIP **(C)**. *Note:* The lowest reportable value for α-MSH is 8 pg/mL, reflecting the detection limit of the clinical assay.

**Figure 3:**
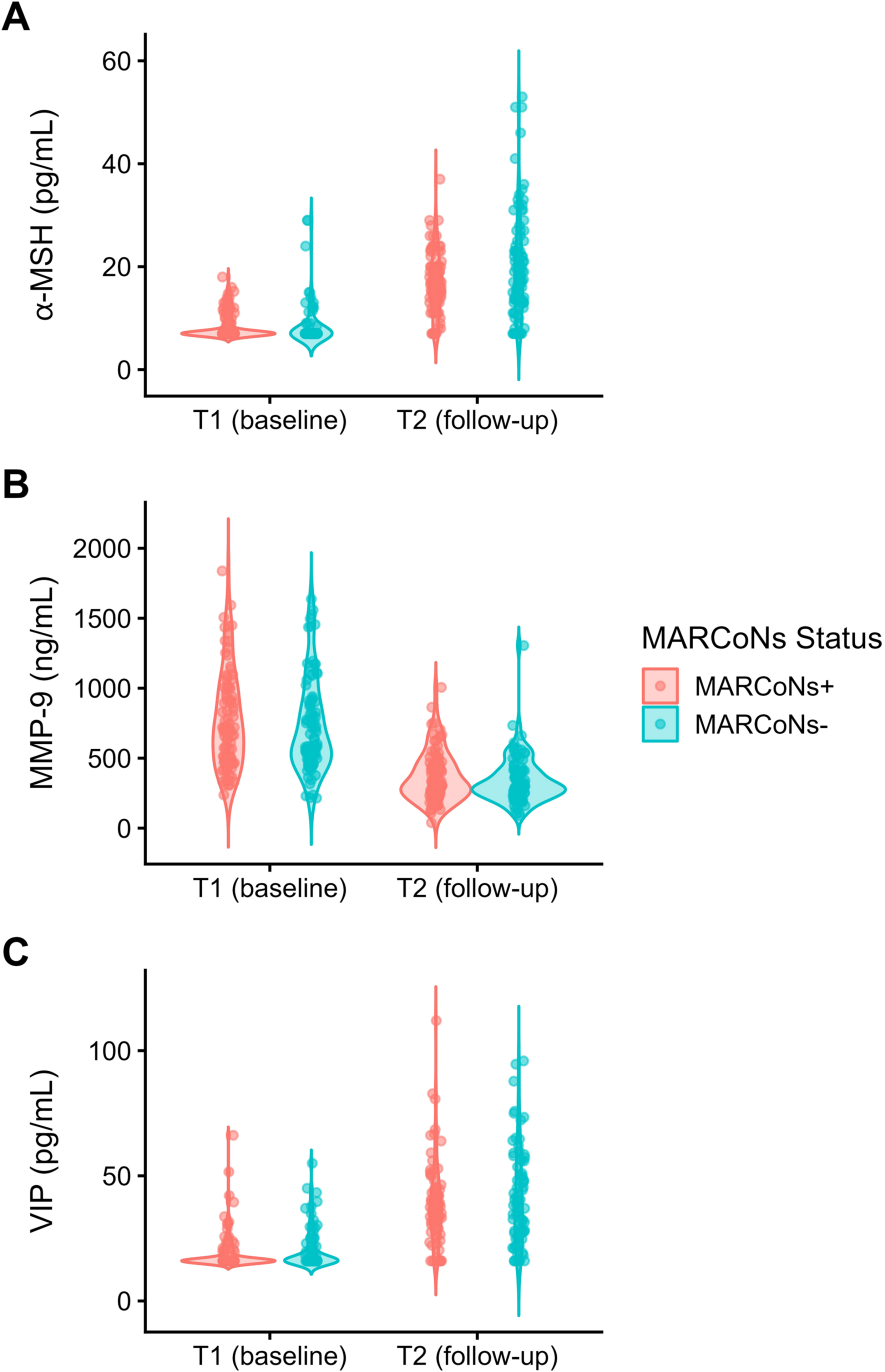
Violin plots depicting observed circulating levels of **(A)** α-MSH, **(B)** MMP-9, and **(C)** VIP at baseline (T1) and follow-up (T2), grouped by follow-up MARCoNS Status (positive vs. negative). Individual patient observations are overlaid. Note: The lowest possible value for α-MSH is 8, based on the detection limit of the laboratory assay used.

As shown in **Table 5** and **Figure 2A** and **3A**, α-MSH exhibited a significant interaction between MARCoNS Status and Timepoint, indicating that longitudinal change differed as a function of follow-up culture status. Follow-up simple effects analyses demonstrated significantly higher α-MSH levels at T2 among patients who were MARCoNS-negative compared with those who remained MARCoNS-positive (β = 3.92, SE = 0.99, df = 345, *t* = 3.93, *p* <.001, 95% CI [1.97, 5.88]). By contrast, no group difference was observed at baseline (β = -0.13, SE = 0.99, df = 345, *t* = -0.13, *p* = .895, 95% CI [-2.09, 1.82]), supporting comparability of α-MSH levels prior to the follow-up interval.

To further evaluate the robustness of this association, the baseline-adjusted regression model (**Table 8**) predicting follow-up α-MSH levels confirmed that MARCoNS status at T2 remained a significant independent predictor of circulating α-MSH. After controlling for baseline (T1) α-MSH levels, age, sex, and baseline VIP and MMP-9, patients who were MARCoNS-negative at T2 continued to exhibit significantly higher α-MSH levels at T2. Baseline (T1) α-MSH was also a significant predictor of T2 levels, whereas baseline VIP and MMP-9 were not significant contributors. Together, these findings indicate that the selective association between MARCoNS clearance and higher α-MSH at follow-up persists even after accounting for baseline biomarker levels and broader neuroendocrine-inflammatory covariation.

**Table 8:** ANCOVA regression predicting follow-up α-MSH (T2).

Fixed effects	Estimate	SE	df	t	p	95% CI
Intercept	19.54	0.81	144	24.14	<.001	[17.94, 21.14]
MARCoNS Status	5.59	1.34	144	4.18	<.001	[2.95, 8.24]
α-MSH at T1	4.24	0.66	144	6.39	<.001	[2.93, 5.55]
MMP-9 at T1	0.51	0.68	144	0.76	.449	[-0.82, 1.85]
VIP at T1	-0.77	0.67	144	-1.15	.253	[-2.11, 0.56]
Age	-0.58	0.69	144	-0.84	.403	[-1.93, 0.78]
Sex	-2.46	1.63	144	-1.51	.132	[-5.68, 0.75]

Follow-up α-MSH (T2) was regressed on MARCoNS status at follow-up, adjusting for baseline α-MSH (T1), age, sex, and baseline VIP and MMP-9. Continuous covariates were z-scored and categorical predictors were contrast-coded (−0.5, +0.5). Wald 95% confidence intervals are shown. Model fit: *F*(6, 144) = 11.25, *p* <.001; adjusted *R²* = .29. This analysis serves as a sensitivity test against regression-to-the-mean effects.

Sensitivity analyses excluding patients (N = 19) who initiated exogenous VIP therapy yielded highly similar results (**Supplementary Table 2** in **Supplementary Materials**). The Timepoint × MARCoNS Status interaction remained significant (β = 4.77, SE = 1.26, df = 167, *t* = 3.77, *p* <.001, 95% CI [2.29, 7.25]), suggesting that the differential α-MSH trajectory by follow-up MARCoNS status was not attributable to VIP use.

Finally, we examined whether MARCoNS status was associated with comparable longitudinal variation across other biomarkers assessed in this cohort. Importantly, no significant MARCoNS Status × Timepoint interactions were observed for MMP-9 (**Table 6**; **Figure 2B** and **3B**) or VIP (**Table 7**; **Figure 2C** and **3C**; **Supplementary Tables 3** and **S4** in **Supplementary Materials**). This pattern suggests that follow-up MARCoNS culture status was selectively associated with longitudinal variation in α-MSH, rather than reflecting a generalized effect across all biomarkers examined.

## Discussion

Researchers have examined the toxigenic and putative membrane-disruptive effects of CoNS strains and their association with persistent symptoms. Butt and colleagues were the first to state that the membrane-damaging toxins formed by CoNS could be responsible for chronic orofacial pain (39). Further work by McGregor and colleagues again found an association between the elevated presence of CoNS and chronic orofacial muscle pain (40). Subsequently, Metcalf and colleagues expanded the research to include orofacial pain in persons with chronic fatigue syndrome (46). Their data suggested that putative membrane-damaging toxins produced by CoNS were associated with orofacial pain with increased prevalence in patients with chronic fatigue syndrome. These studies provided early evidence linking CoNS with chronic pain phenotypes, though mechanistic pathways remain incompletely defined.

The melanocortin system, including α-MSH, has been studied for its anti-inflammatory and pain-modulating actions, largely based on preclinical and translational evidence (7, 8, 11). In this context, the selective association observed in the present study may indicate that α-MSH trajectories track more closely with MARCoNS status than with broader inflammatory markers. However, the biological mechanisms underlying this specificity remain unclear. Future prospective studies incorporating more detailed endocrine phenotyping and symptom-based outcomes (e.g., chronic pain severity) will be needed to determine whether α-MSH represents a clinically meaningful mediator or marker of recovery in patients with chronic inflammatory illness.

Shoemaker and colleagues (37, 38) were the first to document an association between α-MSH deficiency and the presence of MARCoNS in patients suffering from persistent multi-system symptoms. Notably, low circulating α-MSH levels were found in over 93% of symptomatic individuals in that cohort, compared with 3% of control participants. In contrast to MARCoNS-negative patients, who experienced symptom improvement following administration of cholestyramine, patients who tested positive for MARCoNS reported persistent symptom burdens until after initiating antimicrobial treatment of the colonization. The present findings build from these initial clinical observations by providing quantitative longitudinal evidence of the association between MARCoNS and α-MSH. Together, these results suggest that α-MSH may represent a particularly informative neuroimmune regulatory marker in this patient population, where persistent mucosal colonization and immune dysregulation are hypothesized to contribute to ongoing symptom burden and biomarker abnormalities. Prospective mechanistic studies will be required to determine whether α-MSH plays a mediating role in symptom persistence and severity or simply reflects downstream immune normalization.

Our findings also reinforce the importance of distinguishing between bacterial colonization and overt infection, particularly in the context of MARCoNS. While infection involves a breach of host defenses that produces a strong, acute inflammatory signal characterized by tissue destruction and symptomatic illness, colonization reflects the persistence of microbes on mucosal or epithelial surfaces without classical signs of infection (49, 50). Colonization is often a necessary antecedent to infection, but the two processes are mechanistically and clinically distinct.

This distinction is well illustrated by acute infections in which pathogens produce high-amplitude virulence factors that provoke massive cytokine cascades and systemic illness (47, 48, 51, 52). In contrast, MARCoNS represent a colonization functional state rather than an overt invasive infection. Although CoNS are generally regarded as commensals and are not typically associated with classical acute toxin-mediated syndromes, case reports suggest that *S. epidermidis* may, under specific circumstances, harbor enterotoxin/superantigen genes more commonly linked to *S. aureus* (33). However, our retrospective study did not include CoNS speciation or toxin profiling, and thus such possibilities remain untested.

More broadly, CoNS colonization does not imply biological neutrality. CoNS organisms may engage in low-amplitude but persistent pathogenic strategies, including biofilm formation, AMP deactivation, protease and lipase secretion, and localized cytokine activity (34–36). In patients presenting environmentally related multisystem illness, where immune signaling may be disorganized rather than globally suppressed, these colonization strategies could plausibly disrupt neuroimmune homeostasis. Such low-grade, persistent immune activation may represent one possible explanation for why MARCoNS colonization is clinically associated with α-MSH deficiency even in the absence of overt infection.

While the present study was not designed to test causal mechanisms, the observed specificity of MARCoNS status for α-MSH trajectories highlights the need for further research into how persistent sinonasal colonization may relate to systemic neuroimmune regulation. CoNS organisms may influence host physiology through indirect inflammatory signaling, epithelial immune interactions, or other pathways that remain incompletely understood. Future prospective studies incorporating standardized sampling intervals, broader covariate characterization, and mechanistic endpoints will be necessary to determine whether α-MSH functions as a downstream marker of recovery, a mediator of symptom persistence, or both, in chronic inflammatory illness populations.

Given the retrospective observational design and post-treatment stratification by follow-up culture status, residual confounding cannot be excluded in the present study (e.g., differences in baseline illness severity, treatment intensity or adherence, selection biases, and concurrent interventions occurring alongside MARCoNS-directed therapy). Although a substantial proportion of baseline α-MSH values were at the lower assay boundary, the reduction in floor values from T1 to T2 occurred similarly across MARCoNS status groups, indicating that the observed interaction reflects differences in continuous α-MSH levels rather than merely transition above the detection limit. Finally, follow-up intervals between baseline and post-treatment laboratory assessments could not be quantified because specimen collection dates were not available in the retrospective data extract; thus, variability in time-to-follow-up may contribute to outcome heterogeneity and cannot be modeled directly.

## Conclusion

This retrospective observational study provides quantitative evidence that follow-up MARCoNS clearance status is selectively associated with higher circulating α-MSH levels in a clinically characterized cohort evaluated within a CIRS-informed clinical framework. Notably, this association was specific to α-MSH and was not observed for VIP or MMP-9, suggesting that persistent sinonasal colonization may relate to melanocortin-linked regulatory pathways rather than reflecting a generalized biomarker shift. While coagulase-negative staphylococci are often regarded as commensal colonizers rather than acute pathogens, these findings highlight the potential clinical relevance of antibiotic-resistant nasal colonization in chronic multisystem illness populations. Given the observational design and potential residual confounding, prospective studies with standardized sampling intervals and mechanistic endpoints will be needed to clarify whether α-MSH represents a downstream marker of recovery, a mediator of symptom persistence, or both, and to determine how targeted MARCoNS-directed interventions influence longitudinal clinical outcomes.

## Data Availability

The raw data supporting the conclusions of this article will be made available by the authors, without undue reservation.
