# Supplementary material for: Clearance of multiple antibiotic-resistant coagulase-negative staphylococci is selectively associated with higher circulating α-melanocyte stimulating hormone in patients evaluated for chronic inflammatory response syndrome
Source: Front Endocrinol (Lausanne). 2026 Apr 17;17:1728408. doi: 10.3389/fendo.2026.1728408 (PMC13132755; doi:10.3389/fendo.2026.1728408)
Supplement: Supplementary file 1 [file DataSheet1.pdf]

Table S1. Symptom cluster assessment used for clinical evaluation of CIRS

Cluster	Symptoms included
Cluster 1	Fatigue
Cluster 2	Difficulty concentrating
Cluster 3	Weakness; Decreased assimilation of new knowledge; Aches; Headache; Light sensitivity
Cluster 4	Unusual skin sensitivity; Tingling
Cluster 5	Shortness of breath; Sinus congestion
Cluster 6	Memory impairment; Decreased word finding
Cluster 7	Coughing; Excessive thirst; Confusion
Cluster 8	Joint pain; Morning stiffness; Cramps
Cluster 9	Appetite swings; Difficulty regulating body temperature; Increased urinary frequency
Cluster 10	Red eyes; Blurred vision; Night sweats; Mood swings; Ice-pick pain
Cluster 11	Abdominal pain; Diarrhea; Numbness
Cluster 12	Tearing of eyes; Disorientation; Metallic taste
Cluster 13	Static shocks; Vertigo

Note. Symptoms are grouped according to the cluster-based screening instrument commonly used in clinical practice to characterize multisystem symptom patterns. The table is provided for background description of the assessment method and was not used as a primary outcome measure in the present study.

Table S2. Model estimates for α-MSH excluding 19 patients on VIP treatment (N = 169).

Fixed Effects	Estimate	*SE*	df	*t*	*p*	95% CI
Intercept	13.63	0.53	165	25.83	<.001	[12.59, 14.66]
Age	-0.82	0.43	165	-1.90	.060	[-1.67, -0.03]
Sex	-0.36	1.06	165	-0.34	.737	[-2.43, 1.72]
MARCoNS Status	2.34	0.86	167	2.73	.007	[0.66, 4.01]
Timepoint	9.97	0.63	167	15.78	<.001	[8.74, 11.21]
MARCoNS × Timepoint	4.77	1.26	167	3.78	<.001	[2.30, 7.25]

Random Effects	Variance	*SD*
Intercept | Patient	14.00	3.74
Residual	33.73	5.81

Note. df computed using Satterthwaite approximation (lmerTest). Confidence intervals are Wald 95% intervals from confint(). Random effects are reported as variance components for the patient-level random intercept and residual error. Age was z-standardized and categorical predictors were sum-coded (±0.5), such that the intercept represents the grand mean biomarker level across groups and timepoints.

Table S3. Model estimates for MMP-9 excluding 19 patients on VIP treatment (N = 166).

Fixed Effects	Estimate	*SE*	df	*t*	*p*	95% CI
Intercept	548.12	21.00	162	26.11	<.001	[506.97, 589.26]
Age	-23.79	17.15	162	-1.39	.167	[-57.41, 9.83]
Sex	16.43	42.06	162	0.39	.697	[-66.00, 98.87]
MARCoNS Status	-35.84	33.95	162	-1.06	.293	[-102.39, 30.71]
Timepoint	-387.29	22.35	164	-17.33	<.001	[-431.10, -343.21]
MARCoNS × Timepoint	25.65	44.70	164	0.57	.567	[-61.98, 113.27]

Random Effects	Variance	*SD*
Intercept | Patient	26936	164.10
Residual	41407	203.50

Note. df computed using Satterthwaite approximation (lmerTest). Confidence intervals are Wald 95% intervals from confint(). Random effects are reported as variance components for the patient-level random intercept and residual error. Age was z-standardized and categorical predictors were sum-coded (±0.5), such that the intercept represents the grand mean biomarker level across groups and timepoints.

Table S4. Model estimates for VIP excluding 19 patients on exogenous VIP treatment (N = 136).

Fixed Effects	Estimate	*SE*	df	*t*	*p*	95% CI
Intercept	29.49	1.21	132	24.39	<.001	[27.12, 31.86]
Age	2.48	0.99	132	2.50	.014	[0.53, 4.42]
Sex	0.86	2.42	132	0.36	.723	[-3.88, 5.60]
MARCoNS Status	0.51	1.96	132	0.26	.795	[-3.33, 4.35]
Timepoint	18.69	1.25	134	15.01	<.001	[16.25, 21.14]
MARCoNS × Timepoint	0.30	2.49	134	0.12	.904	[-4.58, 5.18]

Random Effects	Variance	*SD*
Intercept | Patient	77.31	8.79
Residual	105.48	10.27

Note. df computed using Satterthwaite approximation (lmerTest). Confidence intervals are Wald 95% intervals from confint(). Random effects are reported as variance components for the patient-level random intercept and residual error. Age was z-standardized and categorical predictors were sum-coded (±0.5), such that the intercept represents the grand mean biomarker level across groups and timepoints.
